# 

**Published:** 1889-12-28

**Authors:** 


### NOTICE TO CORRESPONDENTS.

All MS., Letters, Books for Review, and other matters intended for the Editor, should be addressed The Editor, The Lodge, Porchester Square, London, W.

**Notice to Advertisers and Subscribers.**

All Advertisements, Orders for Copies of The Hospital, and Business Communications should be addressed to The Publisher, *not to the Editor*, The Hospital, 139-140, Salisbury-court, Fleet-street, E.C.

**Bound Volumes of "The Hospital."**

Vol. VI., containing full page portraits of T.R.H. the Prince and Princess of Wales, and Miss Florence Nightingale, is now ready, 4s., post free. Cases for binding, may be had of the Publisher, at 1s. 6d. each.

**To Residents, Secretaries, and Matrons**

The Editor will be glad to have the Regulations and each Report as ssued by Asylums, Hospitals, Convalescent and Nursing Institutions, as well as those of other Charities, and an early copy in duplicate of all Appeals and Festival Notices, etc., addressed to *The Editor, The Lodge, Porchester-square, London, W.* Any superintendent, secretary, matron, or other chief official who regularly sends such information may be registered, on application to the Publisher, to receive free of cost a cover for binding The Hospital in half-yearly volumes.

### Scraps and Gleanings.

Wigan has decided to have an ambulance similar to the Bootle one.

A fete will be held early next year to wipe off the debt of £600 on the Morley House Convalescent Home.

Warrington Hospital Sunday Fund has reached £216. Last year it was £195; this is a considerable increase.

The Duchess of Teck, as president of the Ladies' Needlework Society, has sent a large box of clothing to St. John's Hospital, Twickenham.

The National Society for the Prevention of Cruelty to Children have established a Camberwell and East Dulwich branch of the society.

Up to Dec. 2nd 340 first-year medical students had entered in the University of Edinburgh, of whom 140 began their studies last summer session.

The bazaar lately held in the Town Hall, Kingstown, for the Monkstown Hospital, has realised a sum of £464. This amount will be placed to the credit of the building fund.

The students of Victoria Dental Hospital, Manchester, dined together on the 14th inst., Mr. H. Planck in the chair. Amongst the speakers after dinner were Dr. Parson Shaw and Mr. G. Ream.

The Marquis of Waterford, as chairman of the London committee of the Irish Distressed Ladies' Fund, writes to point out to the public how urgently money is required in support of this charity.

York County Hospital has not benefitted much by the late exhibition; the secretary of the hospital has been writing and publishing severe things about Mr. Joseph Davies, who got up the exhibition.

The next annual festival in aid of the Benevolent Branch of the Dramatic and Musical Sick Fund will take place on Ash Wednesday, February 19, 1890, at the Hotel Metropole. Mr. Henry J. Leslie will preside.

The *London Gazette* contains a new muzzling order extending the regulations at present in force to various boroughs and county districts in Kent, Essex, Surrey, Herts., Lancashire, Cheshire, and the West Riding of Yorkshire.

The Society for the Propagation of the Gospel in Foreign Parts has received a donation of £9,000, in memory of the late Rev. John Turner, for some time a curate of the Parish Church of Whitby. The donation is made by his mother in his memory, and by his special desire.

A loan exhibition of pictures and other works of art in aid of the building fund of the Great Northern Central Hospital is to be opened by the Marchioness of Lorne on Monday, Jan. 6th, 1890, in the gallery of the Camden School of Art, 4, St. Bartholomew-road, Camden-road, N.

Dr. Gairdner, of Glasgow, has lately delivered an address to the Midland Medical Society, in Birmingham, on "Drunkenness and Dipsomania: Medical and Legal Preventives and Remedies." He strongly supported more stringent measures for the compulsory detention of habitual drunkards.

The Prince and Princess of Wales will leave town on Tuesday, January 14th, on their projected visit to Lord and Lady Wimborne, at Canford, in Dorset. Thursday and Saturday will be occupied by the ceremonials associated with the opening of the Victoria Hospital at Bournemouth, and the People's Park at Poole.

In a German pamphlet published in 1833 the following list of names is given as having been applied to the epidemic now raging on the Continent: 1, Anguinaglia; 2, Ladendo; 3, Coqueluche; 4, Burzel; 5, Genser: 5, Malum Castronis; 6, Mal de Castrone; 7, Schafhusten; 8, Huhnerweh; 9, Ziep; 10, Spanischer Pips; 11, La Grippe; and, finally, 12, Influenza.

